# Under the Gaze: How and When Being Observed Facilitates Innovative Behavior

**DOI:** 10.3390/bs16040532

**Published:** 2026-04-01

**Authors:** Xue Zhang, Guyang Tian, Liang Liang, Yezhuang Tian, Zhongqiu Li

**Affiliations:** 1 College of Philosophy, Law and Political Science, Shanghai Normal University, Shanghai 200234, China; xuezinihao@163.com; 2 School of Business and Management, Wuxi Normal College, Wuxi 214000, China; 3 College of Economics and Management, Northeast Forestry University, Harbin 150040, China; liangliang@nefu.edu.cn; 4 School of Management, Harbin Institute of Technology, Harbin 150001, China; 5 College of Economics and Management, Northeast Agricultural University, Harbin 150038, China; lizhongqiu@neau.edu.cn

**Keywords:** social facilitation, innovative behavior, being observed, prevention focus, promotion focus, willingness to share knowledge, regulatory focus theory

## Abstract

Drawing on social facilitation theory and regulatory focus theory, we propose and test a moderated mediation model in which being observed promotes employees’ innovative behavior through willingness to share knowledge, with regulatory focus serving as a key boundary condition. We tested the model in two complementary studies: a field experiment (*N* = 223) and a two-wave survey (*N* = 103). Across both studies, being observed was positively related to willingness to share knowledge, which in turn predicted innovative behavior. In the survey study, prevention focus (an individual’s orientation toward fulfilling duties, responsibilities, and avoiding negative outcomes) strengthened the positive effect of being observed on willingness to share knowledge and magnified the resulting indirect effect on innovative behavior. By contrast, promotion focus (an individual’s orientation toward pursuing aspirations, personal growth, and attaining positive outcomes) attenuated the link between being observed and willingness to share knowledge, although the conditional indirect effect did not reach significance. These findings contribute to the social facilitation literature by specifying the knowledge sharing mechanism and the regulatory-focus boundary conditions through which social attention translates into innovative behavior in organizational field settings.

## 1. Introduction

Innovation has become a core determinant of organizational success in an era of intensifying global competition and rapid technological change (Anderson et al., 2014; Shalley et al., 2004). Individual innovative behavior, which refers to the intentional generation, promotion, and realization of new ideas within a work role, workgroup, or organization (Janssen & Van Yperen, 2004, p. 370), occupies a central place in job performance frameworks and has therefore attracted substantial scholarly attention (Harari et al., 2016; Hughes et al., 2018; Bui et al., 2025; Khan et al., 2025). Because the effectiveness of individual innovation depends largely on both contextual conditions and employee attributes (Anderson et al., 2014; Chen et al., 2016; Zhou & Hoever, 2014), scholars have increasingly examined the social and situational factors that facilitate or constrain the innovation process (Axtell et al., 2000; Ng et al., 2010; Yuan & Woodman, 2010). Among such factors, social influence, particularly the experience of being observed by others, has been identified as a key antecedent of employee behavior (Shalley & Perry-Smith, 2001). Being observed refers to a situation in which an individual is watched while performing an action, and it is widely relevant to modern organizations in which monitoring has become pervasive (Pfattheicher & Keller, 2015).

The practical relevance of this topic has grown substantially in recent years. Survey evidence indicates that approximately 80% of large employers deploy some form of electronic monitoring (American Management Association, 2019), and by 2025 an estimated 70% of large corporations adopted AI-augmented monitoring systems, and AI-enabled monitoring expanded sharply during the shift to remote (Ravid et al., 2023; Yam et al., 2023) and hybrid work following the COVID-19 pandemic (Bernstein et al., 2020; Tursunbayeva et al., 2022). These developments have generated a significant paradox. On the one hand, a substantial body of evidence suggests that employees generally dislike being monitored, and that surveillance can increase occupational stress, reduce perceived autonomy, and erode interpersonal trust (Ravid et al., 2020; Stanton, 2000; Holland et al., 2015). On the other hand, research also demonstrates that monitoring can enhance job performance, increase compliance with organizational norms, and promote prosocial behavior (Bhave, 2014; Claypoole & Szalma, 2019; McNall & Roch, 2009). For instance, Bhave (2014) found that electronic monitoring improved performance among call-center employees, while Bateson et al. (2006) demonstrated that subtle observation cues increased cooperative behavior in a real-world setting. This paradox underscores the need to move beyond the question of whether observation helps or harms employee behavior and instead specify when and how being observed shapes specific forms of workplace behavior. This is particularly important for innovative behavior, which is inherently complex and may respond to social presence in nuanced ways.

Despite the growing salience of workplace observation, theoretical understanding of its influence on innovative behavior remains underdeveloped. Prior experimental research has examined the relationship between being observed and task performance (Bond & Titus, 1983; Steinmetz & Pfattheicher, 2017), yet far less attention has been devoted to creative and innovative outcomes in field settings. Social facilitation theory posits that the presence of others enhances performance on simple, well-learned tasks but may impair performance on complex, novel ones (Zajonc, 1965). Because innovative behavior requires novel idea generation, flexible problem solving, and the willingness to take risks (Henchy & Glass, 1968; Amabile, 2018), the influence of observation on innovative behavior is likely to be more nuanced than the simple facilitation-inhibition dichotomy suggests. More importantly, a specific gap in the literature concerns the mechanism through which observation translates into innovative behavior. Although scholars have speculated that willingness to share knowledge may link social presence to innovation (Lu et al., 2012; J. Wang et al., 2017), no study has empirically tested this mediating pathway in field settings while simultaneously examining individual difference moderators that specify for whom observation is most consequential. This gap is significant because it leaves practitioners without evidence-based guidance on how to design observation systems that support, rather than suppress, employee innovation.

We focus on willingness to share knowledge as the explanatory mechanism for several theoretically grounded reasons. Organizational and interpersonal contexts have long been recognized as key determinants of workplace knowledge-sharing processes (S. Wang & Noe, 2010; Haak-Saheem, 2016; Youssef et al., 2017). When employees perceive that they are being observed, social facilitation theory suggests that they experience heightened arousal and increased evaluation apprehension, which motivate them to display competence and contribute positively to their workgroup (Steinmetz & Pfattheicher, 2017). Knowledge sharing, which refers to acts of making knowledge available to others within the organization (Ipe, 2003, p. 32), represents one prominent avenue through which employees can signal competence and goodwill. Moreover, a substantial body of research has demonstrated that knowledge sharing is a proximal antecedent of innovative behavior at both the individual and team levels (Dong et al., 2017; Yu et al., 2013; Afsar, 2016; J. Wang et al., 2017; Kim & Lee, 2013). By linking being observed to willingness to share knowledge and, in turn, to innovative behavior, we offer a more fine-grained account of the social facilitation process in organizational settings.

To further specify boundary conditions, we integrate regulatory focus theory (Higgins, 1997, 1998) with social facilitation theory. Regulatory focus theory distinguishes between prevention focus and promotion focus. Prevention focus orients individuals toward duties, responsibilities, and the avoidance of negative outcomes, whereas promotion focus orients individuals toward aspirations, growth, and the pursuit of positive outcomes. These self-regulatory orientations shape how individuals respond to social cues and evaluation pressure (Pfattheicher, 2015; Keller & Pfattheicher, 2011). We argue that prevention focus amplifies the effect of being observed on willingness to share knowledge because prevention-focused employees are especially attentive to others’ evaluations and motivated to conform to social expectations. Conversely, promotion focus attenuates this effect because promotion-focused employees are guided more by internal aspirations than by external evaluation cues. By incorporating regulatory focus as a moderator, we clarify for whom observation is most likely to translate into innovative behavior.

This study makes four contributions to the literature. First, it extends social facilitation theory to the domain of innovative behavior by demonstrating that the experience of being observed can foster, rather than uniformly inhibit, complex work behaviors. This finding addresses contradictory evidence in the social facilitation literature regarding the effects of observation on nonroutine tasks (Hills et al., 2019; Uziel, 2007). Second, it identifies willingness to share knowledge as a novel mediating mechanism through which observation influences innovative behavior, thereby bridging the social facilitation and knowledge management literature. Third, by incorporating regulatory focus as a boundary condition, it clarifies for whom being observed is most likely to promote willingness to share knowledge and, ultimately, innovative behavior. This integration responds to recent calls for theory-driven models that simultaneously examine mediators and moderators of observation effects (Steinmetz & Pfattheicher, 2017; Pfattheicher, 2015). Finally, by combining a randomized field experiment with a two-wave survey, this study strengthens both causal inference and external validity beyond the laboratory paradigms that have dominated prior research on social facilitation (Hills et al., 2019).

## 2. Theoretical Background and Hypotheses

### 2.1. Social Facilitation Theory

Social facilitation is an interaction-centered theoretical perspective that emphasizes how social presence shapes individual behavior (Zajonc, 1965). The theory originated from Triplett’s (1898) seminal observation that cyclists pedaled faster when racing alongside others, and it was formalized by Zajonc (1965), who proposed that the mere presence of others increases drive, which in turn enhances the performance of dominant responses and impairs the performance of non-dominant (novel or complex) responses. Subsequent theoretical refinements have invoked evaluation apprehension (Cottrell, 1972; Henchy & Glass, 1968), distraction conflict (Baron, 1986), and self-presentation (Bond, 1982) as complementary explanatory mechanisms for these effects.

In management research, scholars have examined the effects of working in the presence of others on individual performance and productivity through the lens of social facilitation. This approach is appropriate because observer attention can trigger psychological and physiological arousal, which is widely regarded as a key driver of both social facilitation and social inhibition (Bond, 1982; Wicklund & Duval, 1971). Such arousal may lead employees to display either enhanced or diminished performance, depending on task complexity and individual differences (Mullen et al., 1997; Zajonc, 1965; Steinmetz & Pfattheicher, 2017). In the context of employee outcomes, a typical social facilitation process begins with socio-motivational drivers, followed by efforts to seek positive evaluations and avoid negative evaluations from others, and ultimately influences employees’ interpersonal behavior (Steinmetz & Pfattheicher, 2017).

**Figure 1:**
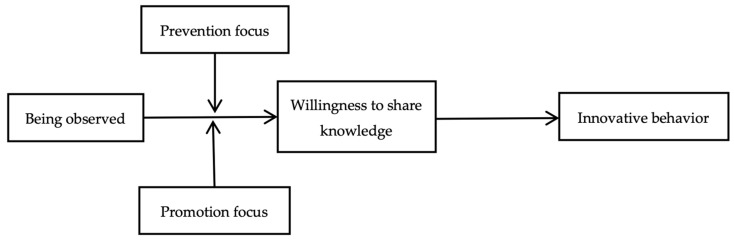
Theoretical Model.

Nevertheless, focusing solely on individuals’ interpersonal behaviors is insufficient to fully capture the social facilitation process, because intrapersonal perceptions and subjective experiences of others’ attention can also vary across individuals (Boothby et al., 2014; Shteynberg et al., 2016; Steinmetz et al., 2016). Accordingly, by integrating insights from social facilitation theory and regulatory focus theory, we hypothesize that being observed influences employees’ innovative behavior through willingness to share knowledge. We further hypothesize that prevention focus strengthens this indirect relationship by amplifying the effect of being observed on willingness to share knowledge and, in turn, innovative behavior. The overall research model is presented in Figure 1.

### 2.2. Being Observed and Willingness to Share Knowledge

A long-standing and robust body of evidence indicates that human behavior is strongly influenced by the mere presence of other individuals (Markus, 1978; Zajonc et al., 1969; Pfattheicher & Keller, 2015). Being observed has been positively associated with desirable organizational and individual outcomes, including verbal learning (Higgs & Joseph, 1971) and job performance (Henchy & Glass, 1968), as well as behaviors such as cooperation, honesty, and generosity (Bateson et al., 2006; Ernest-Jones et al., 2011; Haley & Fessler, 2005; Rigdon et al., 2009). These effects are commonly interpreted through the social facilitation framework, which posits that the social presence of another person can shape human performance (Bond & Titus, 1983).

It is worth noting that being observed can influence multiple domains of employee behavior beyond knowledge sharing. Research has documented effects of observation on task performance (Claypoole & Szalma, 2019), prosocial behavior (Pfattheicher & Keller, 2015), ethical conduct (Bateson et al., 2006), and even counterproductive work behavior such as cyberloafing (Lim, 2002). In addition, observation may affect how employees allocate their time between routine and discretionary activities (Bernstein, 2012). We acknowledge that knowledge sharing is not the only pathway through which observation may shape workplace outcomes. However, we focus on willingness to share knowledge for two specific reasons. First, knowledge sharing is a particularly proximal antecedent of innovative behavior (Dong et al., 2017; Kim & Lee, 2013), making it a theoretically compelling mediator in the observation–innovation link. Second, unlike other potential outcomes of observation, such as task effort or compliance behavior, willingness to share knowledge reflects the interpersonal exchange of information and expertise that is particularly relevant to the idea generation, promotion, and realization stages of the innovation process (Lu et al., 2012).

A firm’s success and competitiveness depend heavily on the effectiveness of its knowledge sharing (Bavik et al., 2018; Davenport & Prusak, 1998). Knowledge sharing is defined as “acts of making knowledge available to others within the organization” (Ipe, 2003, p. 32), and prior research has consistently identified it as essential for organizational effectiveness and for fostering innovation. Through knowledge sharing, organizations can exploit and leverage knowledge-based resources, reduce production costs, enhance team performance, and strengthen innovative capabilities. Given these potential benefits, many organizations invest substantial resources to encourage knowledge sharing (S. Wang & Noe, 2010). However, despite these investments, organizations often struggle to facilitate effective knowledge sharing (Babcock, 2004). One important reason is that organizations frequently overlook the role of organizational and interpersonal contextual factors in shaping knowledge-sharing processes (Carter & Scarbrough, 2001; Voelpel et al., 2005).

Building on social facilitation theory, we contend that being observed constitutes an important contextual cue that can promote willingness to share knowledge in the workplace. Theoretically, when employees are aware that they are being observed, they may engage in proactive impression management to shape peers’ evaluations (Hills et al., 2019). The presence of others can also provide motivation for willingness to share knowledge because social presence heightens employees’ awareness of evaluation and encourages them to demonstrate competence through their work behaviors (Bhave, 2014). In addition, when employees perceive that others are watching them, they are more likely to seek a favorable impression and project a positive self-image. This heightened self-presentation motivation can increase arousal and strengthen their willingness to share knowledge with others (Schnuerch & Gibbons, 2021). These arguments are consistent with recent meta-analytic evidence suggesting that electronic performance monitoring has a small but positive effect on task performance, partly through increased motivation to meet evaluative standards (Ravid et al., 2023). Accordingly, we propose that employees are more likely to demonstrate willingness to share knowledge when they are being observed.

**Hypothesis 1.**  *Being observed is positively related to willingness to share knowledge.*

### 2.3. The Mediating Role of Willingness to Share Knowledge

In workplace settings, employees often accompany peers and observe their task-related behavior. Because a substantial amount of potentially creative work is performed in the presence of others, accounting for the influence of observation is essential for developing a more comprehensive social psychological understanding of innovative behavior. Innovative behavior is a complex set of actions that includes idea generation, idea promotion, and idea realization (Scott & Bruce, 1994; Janssen, 2000). Social psychology research has identified several factors that influence innovative behavior, including mood states, rewards, and self-evaluation (Silvia & Phillips, 2004; Anderson et al., 2014). This literature also suggests that being observed is a particularly influential situational factor that can shape innovative behavior (Amabile, 2018). Mumm and Mutlu (2011) showed that public praise and social comparison may enhance employees’ motivation to innovate, a finding consistent with social comparison theory and social facilitation theory.

We propose that willingness to share knowledge mediates the relationship between being observed and innovative behavior for three theoretically grounded reasons. First, willingness to share knowledge facilitates employees’ idea generation by enabling access to diverse information, perspectives, and expertise (Dong et al., 2017; Kim & Lee, 2013). Within social facilitation processes, the initial arousal stage emerges because being observed heightens employees’ awareness of others’ evaluations (Carver & Scheier, 1981; Henchy & Glass, 1968). We propose that innovative behavior displayed in the presence of others is typically evaluated as a positive outcome. Strong performance in front of an audience is commonly perceived to increase social approval, whereas poor performance under observation may be seen as damaging to one’s social standing (Chib et al., 2018). Consequently, when employees feel observed, they may be motivated to develop learning strategies to strengthen their knowledge and skills, which in turn can enhance their innovative behavior.

Second, innovative behavior, which often involves heuristic processing and is typically complex, difficult, and unfamiliar, may be influenced by the presence of others. Social facilitation research suggests that social presence tends to enhance performance on simple and well-learned tasks but can undermine performance on complex, novel, or unlearned tasks. However, employees who frequently share knowledge are more likely to acquire relevant knowledge resources and to disseminate technical expertise and insights into work-related problems. Such exchanges can stimulate new associations, promote creative thinking, and enhance cognitive stimulation (Mumford et al., 2002; J. Wang et al., 2017). As a result, when employees are in the presence of an audience, they may be more motivated to share information and develop novel and useful ideas than when they are not being observed.

Third, the presence of others can also elicit motivation to avoid deviant behavior and to pursue similarity and harmony. Under observation, employees may be more inclined to share unique information that supports organizational goals. In addition, employees who frequently share knowledge may feel more confident about promoting and realizing new ideas within the organization. This increased confidence can encourage employees under observation to elaborate on ideas and to generate more original concepts (Erez & Nouri, 2010). Accordingly, we advance the following hypothesis.

**Hypothesis 2.**  *Willingness to share knowledge mediates the positive relationship between being observed and innovative behavior.*

### 2.4. The Moderation Effect of Regulatory Focus

Although the social facilitation process is often associated with positive outcomes, employees vary in how they respond to the social pressure created by observation. A meta-analysis by Uziel (2007) showed that personality is an important factor shaping the social facilitation effect. To account for this variability, scholars have highlighted personality differences as meaningful moderators. Regulatory focus theory proposes that individuals pursue goals, seek pleasure, and avoid pain through two distinct self-regulatory strategies, namely promotion focus and prevention focus (Higgins, 1997, 1998; Higgins et al., 2001). Prevention focus reflects a need for safety, emphasizes the fulfillment of duties and responsibilities, and heightens sensitivity to potential loss-related concerns (Brockner & Higgins, 2001). Individuals with a prevention focus tend to define salient goals in terms of avoiding loss and interpret achievement goals as responsibilities. By contrast, promotion focus reflects a need for growth, a desire for advancement, and the pursuit of aspirations. It leads employees to define salient goals in terms of gains or missed gains and to be particularly sensitive to the presence or absence of positive information (Higgins, 1997).

By integrating social facilitation theory with regulatory focus theory, we propose that both prevention focus and promotion focus moderate the relationship between being observed and willingness to share knowledge. Specifically, employees who perceive that they are being observed and who have a strong prevention focus are more likely to develop intentions to share knowledge. Because basic motivational orientations can moderate the influence of social presence on individual behavior (Keller & Pfattheicher, 2011), employees with a pronounced prevention focus tend to be particularly attentive to how they are perceived by others (Pfattheicher, 2015). They are sensitive to negative feedback and to the possibility of failing to meet others’ expectations. Prevention-focused individuals who hold a socially referenced ought self are also more likely to align their thoughts, feelings, and behaviors with those of their colleagues (Markus & Kitayama, 1991). In particular, prevention-focused employees may respond to subtle cues of observation, such as watching eyes, by increasing prosocial behaviors, including willingness to share knowledge (Keller & Pfattheicher, 2011; Pfattheicher, 2015; Steinmetz & Pfattheicher, 2017). Taken together, these arguments support the expectation that prevention focus strengthens the positive relationship between being observed and willingness to share knowledge.

Conversely, employees who perceive that they are being observed and who have a strong promotion focus may be less likely to demonstrate willingness to share knowledge. Consistent with regulatory focus theory, promotion-focused individuals tend to define their ideal self in ways that are less closely tied to concerns about others’ perceptions and expectations. Key features of promotion-focused self-regulation, such as eagerness, an ideal-self orientation, and a focus on personal development and growth, are not conceptually centered on evaluation concerns. Rather, goals such as hopes, wishes, and aspirations primarily reflect individuals’ own internal standards and personal expectations (Higgins, 1997). In this context, employees with a stronger promotion focus are likely to rely less on social reference cues and to place less emphasis on others’ evaluations. As a result, evaluative social attention is less likely to motivate willingness to share knowledge through a promotion-focused lens (Kessler et al., 2017; Pfattheicher, 2015; Schnuerch & Pfattheicher, 2018). Accordingly, we propose the following hypotheses.

**Hypothesis 3.**  *Prevention focus moderates the relationship between being observed and willingness to share knowledge such that the relationship is stronger when prevention focus is high rather than low.*

**Hypothesis 4.**  *Promotion focus moderates the relationship between being observed and willingness to share knowledge such that the relationship is weaker when promotion focus is high rather than low.*

Given the two moderation hypotheses and the premise that regulatory focus shapes individuals’ knowledge-sharing tendencies, regulatory focus is also likely to affect the strength of the indirect relationship between being observed and innovative behavior. This reasoning implies a moderated mediation pattern in which regulatory focus conditions the indirect effect through willingness to share knowledge. Prior research suggests that regulatory focus can facilitate certain stages of the innovation process (Lanaj et al., 2012). However, in the context of organizational innovative behavior, we argue that regulatory focus functions as a critical boundary condition that determines when and for whom being observed translates into innovative behavior. Accordingly, we propose the following hypotheses.

**Hypothesis 5.**  *Prevention focus moderates the indirect relationship between being observed and innovative behavior through willingness to share knowledge such that the indirect effect is stronger when prevention focus is high rather than low.*

**Hypothesis 6.**  *Promotion focus moderates the indirect relationship between being observed and innovative behavior through willingness to share knowledge such that the indirect effect is weaker when promotion focus is high rather than low.*

## 3. Study 1

### 3.1. Method

#### 3.1.1. Participants and Procedure

This study was conducted at a large private service-sector firm in Northeast China, referred to as “CBSWD.” Established in 2013, CBSWD is a comprehensive tourism and resort company specializing in skiing, travel, and related vacation services. The company provides a variety of leisure, recreation, and hospitality offerings to customers and operates across multiple tourism-related business areas. At the time of the study, the company employed more than 1000 individuals distributed across several functional departments, including operations, marketing, human resources, customer service, and business development. Before formal data collection, we visited CBSWD and conducted in-depth interviews with the CEO and employees from multiple departments. These interviews were designed to clarify three aspects of the research setting: the broader study context, the organization’s emphasis on innovation, and how observation occurs within the firm.

To capture the effects of social facilitation in this context, we recruited 300 employees to complete a paper-based questionnaire. Throughout data collection, participants were assured of confidentiality and anonymity. They were also informed that the project examined workplace behavior. All participants were randomly assigned to one of two conditions, namely an observed condition or a control condition. Participants in the observed condition were informed that the company would implement a peer monitoring system and that they could submit messages about any rule-violating behaviors they observed. By contrast, participants in the control condition received no information about rules or peer monitoring. Participants in both conditions then completed measures of willingness to share knowledge, innovative behavior, and demographic characteristics.

Participants who responded incorrectly to the attention-check items were excluded from the analyses. Specifically, the attention check items were embedded within the survey and consisted of instructed response items (e.g., “Please select ‘strongly agree’ for this item”). Participants who failed to follow these instructions were considered inattentive and were removed from the dataset, consistent with recommended practices for ensuring data quality in survey research (Meade & Craig, 2012; Huang et al., 2012). This screening resulted in a final sample of 223 employees. In this sample, 65.02 percent of participants were male. Participants’ ages were primarily 20 to 25 years (70.85%), followed by 30 to 35 years (16.59%) and 46 to 50 years (2.69%). The most common tenure category was three to five years (34.10%). The sample included employees from various departments and hierarchical levels, including entry-level staff, team leaders, and mid-level managers, reflecting a range of job functions within the organization.

#### 3.1.2. Measurements

All scale items were originally developed in English and translated into Chinese using a back-translation procedure (Brislin, 1986). Unless otherwise noted, items were measured on seven-point Likert scales ranging from 1 (“strongly disagree”) to 7 (“strongly agree”).

Willingness to share knowledge. We assessed willingness to share knowledge with the four-item scale developed by Connelly and Kelloway (2003). A sample item is, “I am willing to help others in this organization with expert knowledge.” The Cronbach’s alpha for this scale was 0.90.

Innovative behavior. We measured innovative behavior with the six-item scale developed by Scott and Bruce (1994). A sample item is “Searches out new technologies, processes, techniques, and product ideas.” Cronbach’s alpha was 0.88.

Control variables. We controlled for age, gender, education, and work tenure because prior research suggests that these variables may be associated with willingness to share knowledge and innovative behavior (J. Wang et al., 2017).

### 3.2. Results

#### 3.2.1. Descriptive Statistics

Table 1 reports the means, standard deviations, reliability estimates, and zero-order correlations for Study 1. Being observed was positively related to willingness to share knowledge (r = 0.18, *p* < 0.01) and innovative behavior (r = 0.30, *p* < 0.01), and willingness to share knowledge was positively related to innovative behavior (r = 0.45, *p* < 0.01).

#### 3.2.2. Confirmatory Factor Analysis

To assess the construct validity of the focal variables, we conducted a series of confirmatory factor analyses (CFAs). The results indicated that the hypothesized two-factor model fit the data adequately (χ^2^ = 194.99, df = 86, χ^2^/df = 2.27, CFI = 0.91, TLI = 0.90, RMSEA = 0.08). Following established benchmarks (Hu & Bentler, 1999), the CFI and TLI values meet the commonly recommended threshold of 0.90. The RMSEA of 0.08 falls at the upper boundary of acceptable fit according to Browne and Cudeck (1993), who classify RMSEA values below 0.05 as indicating close fit, values between 0.05 and 0.08 as indicating reasonable fit, and values above 0.08 as indicating mediocre fit. Thus, the model fit is adequate rather than close. Moreover, the two-factor model provided a significantly better fit than the alternative single-factor model, supporting the discriminant validity of willingness to share knowledge and innovative behavior.

#### 3.2.3. Hypotheses Testing

We conducted independent-samples *t*-tests to examine the hypothesized relationships. To test Hypothesis 1, we compared the observed and control conditions. Participants in the observed condition reported higher willingness to share knowledge (M = 3.96, SD = 0.53) than those in the control condition (M = 3.70, SD = 0.85), and they also reported higher innovative behavior (M = 3.81, SD = 0.53) than those in the control condition (M = 3.43, SD = 0.70). In line with Hypothesis 1, the group differences were statistically significant.

To test Hypothesis 2, we estimated a mediation model using PROCESS Model 4 with 5000 bootstrap resamples (Hayes, 2017). Experimental condition (0 = control, 1 = observed) was entered as the independent variable, willingness to share knowledge as the mediator, and innovative behavior as the dependent variable. The indirect effect was significant (coefficient = 0.09, SE = 0.04, 95% CI [0.02, 0.17]), supporting Hypothesis 2.

### 3.3. Discussion

The results of Study 1 indicated that being observed has a significant effect on both willingness to share knowledge and innovative behavior, and that willingness to share knowledge mediates the relationship between observation and innovative behavior. Although the field experiment offered a basis for causal inference, it also involved limitations. First, the experimental manipulation was a one-time treatment, which may not capture the sustained effects of ongoing workplace monitoring. Second, the study did not examine individual differences that might moderate the relationship between being observed and willingness to share knowledge. Third, willingness to share knowledge and innovative behavior were measured concurrently, which limits the ability to establish temporal precedence. To address these concerns, Study 2 employed a two-wave survey design and examined the role of regulatory focus, thereby extending the findings from Study 1.

## 4. Study 2

### 4.1. Method

#### 4.1.1. Participants and Procedure

This study was conducted at a large private service-sector firm in Northeast China with approximately 800 employees, specializing in hotel accommodation, catering, food production and sales, and cultural tourism services. We initially recruited 300 employees and invited them to complete a paper-based questionnaire. Throughout data collection, participants were assured of confidentiality and anonymity. They were also informed that the project examined workplace behavior. The sample included employees from a range of functional departments, including operations, customer service, logistics coordination, finance, and administrative support. Participants held positions ranging from front-line operational staff to team supervisors, department coordinators, and junior managers.

To mitigate common method bias, the survey was administered in two waves. At Time 1, participants completed measures of being observed, willingness to share knowledge, and demographic characteristics. One month later, at Time 2, participants reported their regulatory focus and innovative behavior. In total, 179 employees completed the Time 1 survey and 129 completed the Time 2 survey, yielding a response rate of 72.06%. Participants who provided invalid responses were excluded, resulting in a final sample of 103 employees. In the final sample, 54.36 percent of participants were male. The age distribution was 25 to 30 years (64.07%), 31 to 35 years (11.65%), and 36 to 40 years (3.88%). The largest proportion reported a work tenure of one to three years (29.13%). The majority of participants (78.64%) worked in team-based settings where interdependent tasks required regular interaction and collaboration with colleagues, providing a context in which knowledge sharing is particularly relevant and feasible.

#### 4.1.2. Measurements

Being observed. Being observed was measured with four items adapted from Claypoole and Szalma (2019). Sample items include “The monitoring system in my organization monitors employees’ behavior” and “In my organization, the monitoring system can disclose details of employees’ performance.” The Cronbach’s alpha for this scale was 0.71.

Regulatory focus. Promotion focus and prevention focus were measured using the 18-item scale developed by Lockwood et al. (2002). Sample items include “I frequently imagine how I will achieve my hopes and aspirations” for promotion focus and “In general, I am focused on preventing negative events in my life” for prevention focus. The Cronbach’s alpha was 0.84 for promotion focus and 0.74 for prevention focus.

Willingness to share knowledge. Willingness to share knowledge was measured using the same four-item scale as in Study 1. The Cronbach’s alpha for this scale was 0.70.

Innovative behavior. Innovative behavior was measured using the same six-item scale as in Study 1. The Cronbach’s alpha for this scale was 0.71.

Control variables. Control variables were measured using the same approach as in Study 1.

### 4.2. Results

#### 4.2.1. Confirmatory Factor Analysis

Table 2 presents the CFA results for the measurement models. The hypothesized five-factor model fit the data well (χ^2^ = 69.04, df = 51, χ^2^/df = 1.35, CFI = 0.95, TLI = 0.92, RMSEA = 0.06). Following Browne and Cudeck (1993), an RMSEA value below 0.05 indicates close fit, values between 0.05 and 0.08 indicate reasonable fit, and values above 0.08 indicate mediocre fit. Thus, the RMSEA value of 0.06 for the five-factor model indicates reasonable fit. At the same time, the other fit indices, including a CFI of 0.95 and a TLI of 0.92, exceed the recommended cutoffs of Hu and Bentler (1999), providing additional evidence that the measurement model fits the data adequately. As shown in Table 2, the five-factor model clearly outperformed all alternative models.

#### 4.2.2. Confirmatory Factor Analysis

Table 3 reports the means, standard deviations, and correlations among the Study 2 variables. Being observed was positively correlated with willingness to share knowledge (r = 0.27, *p* < 0.05) and innovative behavior (r = 0.29, *p* < 0.01), but not significantly related to prevention focus (r = 0.02, ns) or promotion focus (r = −0.06, ns).

### 4.3. Hypothesis Testing

Regression analyses indicated that being observed was significantly and positively associated with willingness to share knowledge (b = 0.27, *p* < 0.01; see Table 4), providing support for Hypothesis 1. To test mediation, we estimated a model using PROCESS Model 4 with 5000 bootstrap resamples (Hayes, 2017). The 95% confidence interval for the indirect effect did not include zero (coefficient = 0.07, SE = 0.04, 95% CI [0.01, 0.16]), indicating a significant indirect effect. These findings provide further support for Hypothesis 2.

The interaction between being observed and prevention focus was significantly positive in predicting willingness to share knowledge (b = 0.32, *p* < 0.01; see Table 4). Simple slope analyses showed that being observed was more strongly and positively associated with willingness to share knowledge when prevention focus was high (1 SD above the mean; simple slope = 2.18, *p* < 0.01). When prevention focus was low (1 SD below the mean), the association was weaker (simple slope = 1.60, *p* < 0.01). These results support Hypothesis 3.

The interaction between being observed and promotion focus was significantly negative in predicting willingness to share knowledge (b = −0.24, *p* < 0.05). Simple slope analyses indicated that the positive association between being observed and willingness to share knowledge was weaker when promotion focus was high (simple slope = 1.60, *p* < 0.01) compared with when it was low (simple slope = 2.18, *p* < 0.01). These findings support Hypothesis 4.

We then used PROCESS Model 7 to test the moderated-mediation hypotheses. The analysis results are presented in Table 5. For prevention focus, the indirect effect of being observed on innovative behavior through willingness to share knowledge was significant when prevention focus was high (indirect effect = 0.14, SE = 0.07, 95% CI [0.02, 0.27]) but not when prevention focus was low (indirect effect = −0.01, SE = 0.03, 95% CI [−0.09, 0.04]). The index of moderated mediation was significant (effect = 0.10, SE = 0.05, 95% CI [0.01, 0.22]), supporting Hypothesis 5.

For promotion focus, the conditional indirect effects were not statistically significant at either high or low levels of promotion focus, and the index of moderated mediation included zero (effect = −0.10, SE = 0.08, 95% CI [−0.55, 0.05]). Hypothesis 6 was therefore not supported.

## 5. Discussion

The present research set out to examine how and when being observed promotes employees’ innovative behavior. Drawing on social facilitation theory (Zajonc, 1965) and regulatory focus theory (Higgins, 1997), we developed and tested a moderated mediation model across two complementary field studies. The results from both the field experiment (Study 1) and the two-wave survey (Study 2) converged to support most of our predictions. Being observed was positively associated with willingness to share knowledge, which in turn predicted innovative behavior, confirming willingness to share knowledge as a mediating mechanism. Moreover, prevention focus strengthened the positive association between being observed and willingness to share knowledge and magnified the indirect path linking observation to innovative behavior. By contrast, promotion focus attenuated the link between being observed and willingness to share knowledge, although the conditional indirect effect did not reach significance.

These findings can be interpreted through the theoretical lens of social facilitation theory in conjunction with regulatory focus theory. Social facilitation theory predicts that the presence of others heightens arousal and evaluation apprehension, which motivate individuals to perform behaviors that are likely to elicit positive evaluations (Zajonc, 1965; Cottrell, 1972). Our results are consistent with this prediction: being observed increased willingness to share knowledge, a behavior widely regarded as prosocial and desirable in organizational settings. The mediating role of willingness to share knowledge aligns with the evaluative component of social facilitation, wherein employees under observation channel their heightened motivation into demonstrating competence and goodwill through information exchange. Importantly, this finding extends the traditional social facilitation framework by showing that the arousal and evaluation pressure triggered by observation do not merely enhance simple task performance but can also promote complex interpersonal behaviors that indirectly foster innovation.

Furthermore, the integration of regulatory focus theory explains why the social facilitation effect is not uniform across individuals. Prevention-focused individuals, who are oriented toward fulfilling duties and avoiding negative outcomes (Higgins, 1997), are theoretically more attuned to evaluative cues from observers, making them especially responsive to the motivational pressure created by observation. The significant moderation by prevention focus corroborates Pfattheicher’s (2015) contention that prevention-oriented self-regulation amplifies behavioral responses to subtle observation cues, and extends this logic to willingness to share knowledge and innovative behavior in field settings. Conversely, the weaker moderation by promotion focus is consistent with the theoretical expectation that promotion-focused individuals, guided primarily by internal aspirations rather than external evaluative pressure (Higgins, 1997, 1998), are less susceptible to the social facilitation effect triggered by observation.

The non-significant conditional indirect effect for promotion focus also merits further theoretical consideration. From the perspective of regulatory focus theory, promotion focused employees may be inclined to share knowledge for reasons that extend beyond observational cues, including personal growth, self-expression, and the inherent value of idea exchange (Higgins, 1997). This may reduce the incremental influence of observation on their willingness to share knowledge. In addition, the relatively small sample size in Study 2 may have constrained the statistical power required to detect a modest conditional indirect effect. Future research with larger samples is needed to better clarify the role of promotion focus in the link between observation and innovation.

### 5.1. Theoretical Implications

This study yields several theoretical implications that advance understanding at the intersection of social facilitation theory, knowledge management, and regulatory focus theory.

First, our findings contribute to social facilitation theory by demonstrating that observation effects extend beyond simple task performance to encompass complex, creative work behaviors. Zajonc’s (1965) original formulation predicted that social presence would impair complex task performance because heightened drive strengthens dominant responses on novel tasks. Our results suggest a more nuanced pattern. Although innovative behavior is inherently complex, social facilitation can still exert a positive effect when it operates through an intermediary mechanism such as willingness to share knowledge. By channeling the increased arousal and evaluation apprehension into prosocial knowledge exchange, employees effectively convert a potentially inhibiting force into a catalyst for innovation. This finding aligns with more recent extensions of social facilitation theory that emphasize evaluative and self-presentational motives rather than mere drive (Bond, 1982; Steinmetz & Pfattheicher, 2017), and helps reconcile contradictory findings in the literature regarding the effects of others’ presence on complex tasks (Hills et al., 2019; Uziel, 2007).

Second, this study bridges the social facilitation and knowledge management literatures by identifying willingness to share knowledge as a mediating mechanism linking observation to innovative behavior. Although scholars have long recognized knowledge sharing as a proximal antecedent of innovation (Dong et al., 2017; S. Wang & Noe, 2010; Kim & Lee, 2013), prior work has not examined the social contextual cues that motivate knowledge sharing in observed settings. Our theoretical model and empirical findings suggest that social presence, as conceptualized in social facilitation theory, is an important contextual factor that can stimulate employees’ willingness to share knowledge. This integration offers a new direction for knowledge management research by highlighting the role of interpersonal visibility and evaluation pressure in shaping knowledge exchange. It also responds to calls in the knowledge management literature for greater attention to the situational antecedents of willingness to share knowledge (S. Wang & Noe, 2010; Youssef et al., 2017).

Third, by incorporating regulatory focus as a boundary condition, our study deepens the theoretical integration between social facilitation theory and individual difference frameworks. Prior research on observation effects has noted that personality moderates social facilitation (Uziel, 2007), but few studies have specified which personality dimensions matter and why. Our findings show that regulatory focus, particularly prevention focus, offers a theoretically grounded explanation for individual differences in responses to observation. This result extends Pfattheicher’s (2015) work on prevention focus and reputational concerns by demonstrating that the interaction between observation and prevention focus has downstream consequences for willingness to share knowledge and innovative behavior. More broadly, the integration of regulatory focus theory into the social facilitation framework suggests that researchers should attend to motivational orientations, not just task characteristics, when predicting how social presence will influence employee behavior.

Finally, by drawing on two field studies, including a randomized field experiment, this research strengthens the causal and ecological validity of the findings beyond the laboratory paradigms that have dominated social facilitation research. By demonstrating that observation effects operate in actual workplace settings with real employees performing their regular duties, our study addresses longstanding calls for greater external validity in the social facilitation literature (Hills et al., 2019; Steinmetz & Pfattheicher, 2017).

### 5.2. Practical Implications

This study offers actionable guidance for organizations seeking to harness observation as a tool for promoting positive employee behaviors. Three practical recommendations follow.

First, organizations can adopt strategies that strengthen employees’ prevention focus in observed contexts. Managers may reinforce prevention focus by implementing monitoring mechanisms, such as electronic performance monitoring, that make evaluation cues more salient while simultaneously emphasizing the importance of meeting team responsibilities and avoiding errors. Such mechanisms can encourage employees to proactively adjust their work processes, which may ultimately support innovation. However, organizations should be mindful that excessive surveillance can trigger stress and resentment (Ravid et al., 2020; Holland et al., 2015), and should therefore calibrate the intensity of monitoring to avoid undermining employee well-being. Practical guidelines include communicating the purpose and scope of monitoring transparently, providing employees with voice in how monitoring systems are designed, and using monitoring data constructively for professional development rather than punitive purposes (Ravid et al., 2023; Tursunbayeva et al., 2022).

Second, given the mediating role of willingness to share knowledge, organizations aiming to strengthen innovative behavior should implement strategies that reliably encourage willingness to share knowledge. When managed appropriately, observation can be used to prompt employees to generate and communicate creative ideas. In particular, organizations can combine observation practices with skill development opportunities by using monitoring feedback to inform training and coaching (Kim & Lee, 2013). Such opportunities can help employees refine their capabilities while also reinforcing their willingness to share knowledge, thereby indirectly promoting innovative behavior.

Finally, organizations may leverage employee monitoring technologies to enhance work-related outcomes. However, when monitoring is implemented improperly, it can generate social pressure that may lead to negative employee responses. It is therefore critical for organizations to recognize the risks, limitations, and perceived invasiveness of monitoring practices. Organizations should ensure that employees can engage in learning and development activities in monitored environments without feeling undue pressure. Observation should be implemented in ways that minimize the risk of inhibiting creativity, particularly when creativity is central to organizational performance.

### 5.3. Limitations and Future Research Directions

Several limitations of this study should be acknowledged, which also point to productive directions for future research. First, this study relied on employees’ self-reported measures to assess willingness to share knowledge and innovative behavior, which may increase the risk of common method bias. Although we mitigated this concern in Study 2 by using a two-wave design that temporally separated the measurement of the independent variable and mediator from the dependent variable, a stronger approach would incorporate supervisor-rated or objective measures of innovative behavior. Future studies should replicate our findings using multi-source data to reduce potential reporting biases.

Second, although prior research has suggested that task characteristics may help explain the relationship between observation and innovation, this study did not include task-related variables in the model. Social facilitation theory predicts that the effects of observation depend on task complexity (Zajonc, 1965). Future research should consider task difficulty alongside the mediators examined here to clarify additional processes through which observation may influence employees’ innovative behavior. Incorporating task characteristics would enable a more comprehensive understanding of the factors shaping the observation–innovation relationship.

Third, the current model examined only one boundary condition, namely regulatory focus. Other individual difference variables, such as self-efficacy, core self-evaluations, stress reactivity, and openness to experience, may also moderate the effects of observation (Uziel, 2007). Beyond individual factors, contextual variables such as leader-member exchange, organizational climate for innovation, and reward systems may shape how observation translates into innovative behavior (Schuh et al., 2018; Youssef et al., 2017). Future research could incorporate these variables to develop a more comprehensive model of observation effects in organizations.

Fourth, both studies were conducted in Chinese service-sector firms, which may limit the generalizability of the findings to other cultural and industrial contexts. Cultural values regarding face, collectivism, hierarchical authority, and harmony orientation may influence how employees respond to observation (Markus & Kitayama, 1991). Employees in collectivist cultures may be more sensitive to cues of social evaluation, which may in turn strengthen the relationship between observation and willingness to share knowledge. Future research should examine the proposed model across different national cultures and industries to assess the boundary conditions and generalizability of the findings.

Fifth, the relatively modest sample size in Study 2 may have limited statistical power, particularly for detecting the conditional indirect effect involving promotion focus. Replication with larger samples is warranted to provide more definitive evidence regarding the moderating role of promotion focus. In addition, future studies might employ experience sampling methods or diary designs to capture within-person fluctuations in the experience of being observed and their real-time effects on willingness to share knowledge and innovative behavior.

## Figures and Tables

**Table 1:** Means, Standard Deviations, and Correlations among Variables in Study 1.

	Mean	SD	1	2	3	4	5	6
1. Gender	0.87	0.70						
2. Age	2.15	0.62	−0.07					
3. Education	2.54	0.69	0.16 *	−0.32 **				
4. Work tenure	3.43	1.10	0.30 **	0.35 **	−0.10			
5. Being observed	0.48	0.50	−0.01	−0.11	0.02	−0.59 **		
6. Knowledge sharing	3.83	0.72	−0.23 **	−0.03	−0.02	−0.14 *	0.18 **	
7. Innovative behavior	3.62	0.66	−0.36 **	0.07	−0.06	−0.12	0.30 **	0.45 **

Note. *N* = 223. * *p* < 0.05, ** *p* < 0.01 (two-tailed).

**Table 2:** Model Fits of Measurement Models in Study 2.

Models	χ^2^	df	χ^2^/df	CFI	TLI	RMSEA
Five-factor model ^a^	69.04	51	1.35	0.95	0.92	0.06
Four-factor model ^b^	83.58	55	1.55	0.92	0.88	0.07
Three-factor model ^c^	121.42	58	2.09	0.82	0.76	0.11
Two-factor model ^d^	126.89	60	2.11	0.81	0.75	0.11
Single-factor model ^e^	231.73	61	3.79	0.51	0.38	0.17

^a^: In this model, all items were influenced by their own factors respectively. ^b^: In this model, items for prevention focus and promotion focus were influenced by the same factor, and items for other variables were influenced by their own factors respectively. ^c^: In this model, items for prevention focus and promotion focus were influenced by the same factor; willingness to share knowledge and innovative behavior were influenced by the same factor. ^d^: In this model, items for prevention focus and promotion focus were influenced by the same factor; being observed, willingness to share knowledge and innovative behavior were influenced by the same factor. ^e^: In this model, there is only one factor influencing all variables.

**Table 3:** Means, Standard Deviations, and Correlations among Variables in Study 2.

Variable	Mean	SD	1	2	3	4	5	6	7	8
1. Gender	0.46	0.50								
2. Age	2.03	0.75	−0.027 **							
3. Education	2.52	0.70	0.24 *	−0.42 **						
4. Tenure	2.83	1.09	−0.36 **	0.51 **	−0.31 **					
5. Being Observed	3.86	0.44	−0.14	0.15	−0.13	0.08				
6. Willingness to share knowledge	4.11	0.28	−0.07	−0.04	−0.04	−0.04	0.27 **			
7. Innovative behavior	3.78	0.44	−0.11	0.21 *	−0.08	0.05	0.29 **	0.32 **		
8. Prevention focus	4.66	0.80	−0.10	0.10	−0.08	−0.08	0.02	0.06	0.04	
9. Promotion focus	5.18	0.77	−0.10	−0.01	0.12	−0.13	−0.06	−0.06	0.10	0.40 **

Note. *N* = 103. * *p* < 0.05, ** *p* < 0.01 (two-tailed).

**Table 4:** Regression Results in Study 2.

Variables	Willingness to Share Knowledge				Innovative Behavior				
	M1	M2	M3	M4	M5	M6	M7	M8	M9
Gender	−0.09	−0.06	−0.05	−0.07	−0.08	−0.05	−0.04	−0.03	−0.01
Age	−0.06	−0.09	−0.14	−0.04	0.24	0.22	0.24	0.17	0.26
Education	−0.06	−0.04	−0.09	−0.02	0.01	0.03	0.04	−0.03	0.03
Tenure	−0.06	−0.05	−0.02	−0.10	−0.10	−0.09	−0.08	−0.07	−0.09
Being observed		0.27 **	0.20 *	0.21 *		0.26 **	0.19 ^+^	0.13	0.14
Willingness to share knowledge							0.28 **	0.15	0.24 *
Prevention focus			0.01					−0.06	
Promotion focus				0.03					0.20 *
Being observed × Prevention focus			0.32 **					0.39 **	
Being observed × Promotion focus				−0.24 *					−0.24 *
R^2^	0.01	0.08	0.18	0.13	0.05	0.12	0.19	0.32	0.25
△R^2^	0.01	0.07 **	0.10 **	0.05 ^+^	0.05	0.07 **	0.07 **	0.13 **	0.06 *

Note. *N* = 103. ^+^ *p* < 0.1, * *p* < 0.05, ** *p* < 0.01 (two-tailed).

**Table 5:** Conditional Indirect Effects in Study 2.

Moderator	Level	Effect	SE	95% CI
Prevention focus	Low	−0.01	0.03	[−0.09, 0.04]
	High	0.14	0.07	[0.02, 0.27]
	Index of moderated mediation	0.10	0.05	[0.01, 0.22]
Promotion focus	Low	0.12	0.06	[−0.01, 0.24]
	High	−0.01	0.06	[−0.12, 0.10]
	Index of moderated mediation	−0.10	0.08	[−0.55, 0.05]

## Data Availability

The data supporting the findings of this study are available from the Corresponding author (Guyang Tian, tianguyang@wxnc.edu.cn) upon reasonable request.
